# A novel adaptive momentum method for medical image classification using convolutional neural network

**DOI:** 10.1186/s12880-022-00755-z

**Published:** 2022-03-01

**Authors:** Utku Can Aytaç, Ali Güneş, Naim Ajlouni

**Affiliations:** 1 Computer Engineering Department, Faculty of Computer Engineering, Istanbul Aydın University, Besyol, Inonu Cd. No: 38, 34295 Kucukcekmece, Istanbul, Turkey; 2 Computer Engineering Department, Istanbul Aydin University, Istanbul, Turkey; 3 Computer Engineering Department, Istanbul Atlas University, Istanbul, Turkey

**Keywords:** Adaptive momentum methods, Nonconvex optimization, Backpropagation algorithm, Convolutional neural networks, Medical image classification

## Abstract

**Background:**

AI for medical diagnosis has made a tremendous impact by applying convolutional neural networks (CNNs) to medical image classification and momentum plays an essential role in stochastic gradient optimization algorithms for accelerating or improving training convolutional neural networks. In traditional optimizers in CNNs, the momentum is usually weighted by a constant. However, tuning hyperparameters for momentum can be computationally complex. In this paper, we propose a novel adaptive momentum for fast and stable convergence.

**Method:**

Applying adaptive momentum rate proposes increasing or decreasing based on every epoch's error changes, and it eliminates the need for momentum hyperparameter optimization. We tested the proposed method with 3 different datasets: REMBRANDT Brain Cancer, NIH Chest X-ray, COVID-19 CT scan. We compared the performance of a novel adaptive momentum optimizer with Stochastic gradient descent (SGD) and other adaptive optimizers such as Adam and RMSprop.

**Results:**

Proposed method improves SGD performance by reducing classification error from 6.12 to 5.44%, and it achieved the lowest error and highest accuracy compared with other optimizers. To strengthen the outcomes of this study, we investigated the performance comparison for the state-of-the-art CNN architectures with adaptive momentum. The results shows that the proposed method achieved the highest with 95% compared to state-of-the-art CNN architectures while using the same dataset. The proposed method improves convergence performance by reducing classification error and achieves high accuracy compared with other optimizers.

## Introduction

In recent years, developments of Deep Neural Networks (DNNs) have combined with large amounts of medical images allows more accurate and rapid diagnosis of disorders. It’s helping neuro-oncologists diagnose patients more accurately and recommend effective treatments. The challenge of early diagnosis of disorders increased the importance of new deep learning techniques in medical science. Convolutional neural networks (CNNs) are the most popular deep learning algorithm in computer vision. The main advantage of CNN compared to traditional networks is that it automatically detects significant features, and the network architecture gives CNN the ability to learn complicated features from images [1]. The new methods are also improving the efficiency of CNNs and their accuracy. In the study [2], the author improved the CNN model by using adaptive dropout instead of the global average pool to perform multi-label classification on the data set of X-ray images. The x-ray images reveal multiple diseases, which makes the problem a multi-label classification problem. They have implemented the multi-label classification problem by splitting it into multiple binary problems and reported improved results. Li et al. [3] addressed the challenge of a small dataset, weak annotations, and varying scales of interest by scaling features extracted from medical images to different sizes to capture the scale-invariant patterns. To detect invariant patterns from features of different scales, they have used the shared kernels. Then they have applied the top-k pooling to extract the highest activations from each feature map in each convolution channel. Wei et al. [4] proposed two-channel CNN for classification of MHSI (Medical Hyperspectral Image) data, including the end-to-end CNN for the extraction of global representative features from unlabeled data and a basic CNN to preserve the information of local details. Agrawal et al. [5] proposed a metric to test the suitable CNN architecture to implement transfer learning. They have performed the classification of gastro-intestinal tract images and compared the performance of five different CNN architectures. In the study [6], the author presented an approach to automatically adjust CNN architecture to increase accuracy and decrease test runs. They have used three optimization algorithms, including GA (Genetic Algorithm), BOA (Bayesian Optimization Algorithm), and NM (Nelder-Mead) in their approach to tune CNN. Finally, the process of creating a fine-tuned CNN architecture using optimization algorithms evaluated over the five thousand biomedical case images belonging to six different classes and results are significant with all three optimization algorithms. In addition to the different implementation approaches of CNN a recent study [7] evaluated the CNN and transfer learning for natural medical images and classified them under the defined set of labels. They have concluded that we can transfer knowledge from natural images to medical images, but if the databases are huge, then the results may vary. The CNN model produces significant results when applied to image processing. Rather than designing new models, the researcher [8] applied a Meta-heuristic optimization algorithm to boost the performance of CNN for medical image classification. Another study by [9] optimized the CNN model for histopathological image classification and reported the significant performance of the model. They have followed UED (uniform experimental design) and performed the parameter optimization of breast cancer histopathological images. Automatic identification of diseases is a great contribution in the field of medicine. Therefore, the author in [10] performed classification using CNN to classify bone scintigraphy images. They also compared the well-known CNN architectures for image classification, including GoogleNet, VGG16, and the ResNet50.

Furthermore, as the current world situation resulting from Covid-19 worsens day by day, researchers are also focusing on the automatic detection of disease using deep learning models. For example, [11] the authors performed classification using a CNN model on 165 x-ray images of Covid-19 patients. They have trained the model with the x-ray images for both positive and negative Covid-19 patients. The results were promising in detecting the disease using the model, which can positively contribute to the recent pandemic.

In recent years, AI applications of medical image analysis have increased [12–21]. For example, the author [12] has introduced brain tumor classification using CNN’s while still some researchers using support vector machines [13], region augmentation [15, 19], wavelet transformations [16, 17]. Although brain tumor image segmentation plays a vital role in [14] and [18], it is out of the scope of this research. As per the recent trend of modeling CNN for medical image classification, the authors of studies [12, 16], and [19] both performed classification using medical images. However, in [19] classified the images into five categories related to 3 brain tumor types by training the parameters of CNN rigorously. While the authors [20] used a new machine learning architecture (CapsNet) in brain tumor classification, there is still time to achieve it due to the slow learning process. In [21], the author classified the 2D-CE-MRI images into three types of brain tumors by applying the transfer learning-based fine-tuning approach.

Stochastic gradient descent (SGD) is considered one of the most efficient algorithms [22] for optimizing CNNs. This optimization method minimizes the objective function J(θ) by controlling the model’s parameter θ ∈ R^d^. The control is attained by updating the parameters in the opposite direction of the gradient of objective function ∇θJ(θ) in comparison to different parameters [23]. The local minimum is represented by the size and number of steps required by the learning rate η of the algorithm. The benefit of the gradient descent method for CNN optimization is its capability to solve multi-oriented complex problems that conventional statistical methods cannot solve. Although it is an efficient algorithm, some limitations degrade its performance, including a high steady error rate and slow convergence. These limitations can be mitigated using a method known as the momentum technique. This technique decreases the steepest descent error. Moreover, it aids in increasing the convergence rate. The drawback of the momentum technique is that when fixed momentum is in a negative gradient direction, it will not arrange the weight down the slope but rather to the error surface [24–26]. The backpropagation (BP) technique computes this gradient that SGD uses, and BP can resolve this issue by helping to extend the fixed momentum to adaptive momentum. This may be achieved by superior adaptation with the iterations while gaining optimal convergence speed. During the iteration process, the adaptive momentum will update itself step by step. The only variable on which this adaptive behavior depends is the prediction of an output value error in each iteration.

This paper presents a novel adaptive momentum technique with two significant benefits: Reducing overall error, achieving the highest accuracy, and accelerating fast convergence. Taken results from performance comparison analysis (confusion matrix) proposed adaptive momentum have improved performance and increased efficiency compared to traditional stochastic gradient descent and other state-of-the-art optimization algorithms. The contributions of this paper can be summarized as follows:

- Proposed boosted backpropagation method for not only for binary classification but also multi-label and multi-class classification, which is more complex and challenging when compared with simple binary classification. The proposed method tested on 3 different medical image datasets [27–29] and achieved highest results.
- Demonstrated how adaptive momentum improves the performance and convergence speed of SGD in CNNs when detecting medical disorders
- Compared adaptive momentum optimization algorithm with other optimizers and performed promising results
- Investigated the performance of pre-trained CNNs when using adaptive momentum, not only against state of the art architectures but also against CNNs trained from scratch using medical imaging data.

The layout of this paper is as follows: The proposed approach is given in The proposed approach. The experimental settings, fine tuning, dataset preprocessing is shown in Experimental study. The experimental results and comparison that show the performance of the proposed method are shown in Evaluation results, and the discussion and future work discussion is presented in Discussion.

## The proposed approach

There is a proportional increase in computational effort when using multilayered networks to compute a wide range of Boolean functions [30]. Using gradient descent, the backpropagation algorithm searches for the minimum of the error function in weight [31, 32]. Learning problems are solved by combining the weights, minimizing the error function. Backpropagation entails both backward and forward steps. It performs a backward pass by adjusting the model’s parameters to minimize the error function [33]. In the forward process, “c” represents the inputs to the neural network with “x” neurons. w_xk_ is the weight of interconnection between the hidden layer and neurons. “k” represents the hidden-layer neurons. The hidden layer can be defined as:

1 $${\text{H}}\left( {\text{k}} \right) = \mathop \sum \limits_{{{\text{x}} = 1}}^{{\text{N}}} {\text{c}}_{{\text{x}}} {\text{w}}_{{{\text{xk}}}} + {\text{b}}_{{\text{h}}}$$

where ${\rm{b}}_{\rm{h}}$ is a bias input layer. In the next step, this hidden layer is passed through an activation function [22]. After calculating the overall output by multiplying the output of the hidden layer neurons with the hidden layer weights ${\rm{w}}_{\rm{xk}}$, the results, pass through an activation function. The aim is to minimize the loss function (ω) by adjusting weights to reach a global minimum; this can be described by the following update rule:

2 $$\omega \to - \omega + \eta \nabla {\text{E}}\left( \omega \right)$$

3 $$\nabla {\text{E}}\left( \omega \right) = \left( {\frac{{\partial {\text{E}}}}{{\partial \omega_{1} }},\frac{{\partial {\text{E}}}}{{\partial \omega_{2} }}, \ldots ,\frac{{\partial {\text{E}}}}{{\partial \omega_{{\text{n}}} }}} \right)$$

to get the gradient of *E* with respect to the ${\text{w}}_{{{\text{pq}}}}$, we use the chain rule;

4 $$\frac{{\partial {\text{E}}}}{{\partial {\text{w}}_{{{\text{pq}}}} }} = \sum\limits_{{\text{k}}} {\frac{{\partial {\text{E}}}}{{\partial {\text{H}}\left( {\text{k}} \right)}}\frac{{\partial {\text{H}}\left( {\text{k}} \right)}}{{\partial {\text{w}}_{{{\text{pq}}}} }}} = \sum\limits_{{\text{k}}} {({\text{q}}_{{\text{k}}} \left( {\text{z}} \right)\tau - {\text{p}}\left( {\text{y}} \right)){\text{c}}_{{\text{p}}} \delta_{{{\text{kq}}}} }$$

The gradient of the error function E is

5 $${\text{E}}_{{\text{W}}} \left( {\text{W}} \right) = \left( {{\text{E}}_{{{\text{w}}_{0} }}^{{\text{T}}} \left( {\text{W}} \right),\;{\text{E}}_{{{\text{w}}_{1} }}^{{\text{T}}} \left( {\text{W}} \right), \ldots ., {\text{E}}_{{{\text{w}}_{{\text{n}}} }}^{{\text{T}}} \left( {\text{W}} \right)} \right)^{{\text{T}}}$$

Which is:

6 $$\frac{\partial E}{{\partial {\text{w}}_{{{\text{xk}}}} }} = {\text{c}}_{{\text{x}}} \left( {{\text{q}}_{{\text{i}}} \left( {\text{z}} \right) - {\text{p}}\left( {\text{x}} \right)} \right)$$

In this case the initial W^0^, the iterative increment formula for the weights takes the form

7 $${\text{w}}\left( {{\text{n}} + 1} \right) = {\text{W}}_{{\text{n}}} - \eta {\text{E}}_{{\text{W}}} \left( {{\text{W}}_{{\text{n}}} } \right)$$

where ƞ > 0 is the learning rate which indicates how far to go along the negative direction of the gradient. However, in this case, the convergence speed is very slow due to the saturation behavior of the activation function in the network, which is even much worse for the network with multi-hidden layer networks [34]. This is because even if the output unit saturates the corresponding decent gradient takes a small value, even if the output error is large, which will result in no significant progress in the weight adjustment. The second disadvantage of this method is the difficulty in choosing a proper learning rate ƞ to achieve fast learning while maintaining the learning procedure stable [35]. These problems contribute to the lack of an inability to apply conventional BP to a wide number of applications.

Momentum term prevents search deviation by observing two successive gradient steps to control or uphold the second. The momentum term is a fraction of the previous weight correction. During the last few years different modified versions of BP versions introduced in most of the work was concerned with the effect of both momentum and learning rates in relation to the speed of conversions. This is because these two parameters have a direct relation to conversion underdamped oscillation conditions. This is usually achieved by modifying Eq. (7) by adding a fraction of the previous weight adjustment, which leads to

8 $${\text{W}}_{{{\text{n}} + 1}} = {\text{W}}_{{\text{n}}} - \eta {\text{E}}_{{\text{W}}} \left( {{\text{W}}_{{\text{n}}} } \right) + \alpha \left( {{\text{W}}_{{\text{n}}} - {\text{W}}_{{{\text{n}} - 1}} } \right)$$

In this case $\Delta {\text{W}}_{{{\text{n}} - 1}} = \left( {{\text{W}}_{{\text{n}}} - {\text{W}}_{{{\text{n}} - 1}} } \right)$, the above equation can now be rewritten as;

9 $$\Delta {\text{W}}_{{\text{n}}} = - \eta {\text{E}}_{{\text{W}}} \left( {{\text{W}}_{{\text{n}}} } \right) + \alpha \Delta {\text{W}}_{{{\text{n}} - 1}} \;\;{\text{n}} = 0, 1, \ldots$$

where $\alpha \Delta {\text{W}}_{{{\text{n}} - 1}}$ is the momentum term while $\alpha$ is the momentum coefficient which is a positive number and $(0 < \alpha < 1)$.

### Backpropagation with adaptive momentum

In conventional BP the use of constant learning and momentum terms is an effective way to accelerate the learning convergence by adjusting these terms during the training process. The use of a small learning rate induces a small change in the network weights from one iteration to the next leading to a smoother learning curve. However, using a larger learning term value would result in a larger change in the network weights, which may cause network instability and oscillatory effect. Suitable momentum coefficient and learning rates are required to achieve fast and stable convergence during the training process. This study intends to introduce a BP algorithm with a variable adaptive momentum coefficient and learning rate. The proposed variable momentum is given by equation as:

10 $$\alpha \left( {\text{n}} \right) = \frac{\beta }{{1 + \exp \left( { - \left| {1 \div \sqrt {E({\mathfrak{n}} \times {\text{E(}}{\mathfrak{n}} - 1{\text{)}})} } \right|} \right)}}$$

where $\beta$ is the forgetting factor $(0 \ll \beta < 1)$.

The initial value of β is expected to be large enough; this will result in the term $1 - \beta^{{\text{n}}}$ close to unity. As such, the initial value of α(n) will be relatively large. It is expected that a rapid convergence of the updated weights can be achieved through a minimal number of iterations, which will be enhanced further as the value of momentum becomes smaller. Hence, it provides low-error performance for the weights update in (7). The momentum tracks of the error E(n) in each epoch and decreases or increases within a given range. We create a velocity variable to store our momentum for every parameter.

The gradient of the error function (3) with respect to W and V (velocity) and given the initial weights ${\text{w}}_{0}$, ${\text{w}}_{1}$, and ${\text{v}}_{0}$, ${\text{v}}_{1}$, the momentum algorithm updates the weights w and v iteratively by;11where $\alpha \in \left( {0,1} \right)$ is the variable adaptive momentum coefficient given by Eq. (10), and $\eta \in \left( {0,1} \right)$ is the learning rate (0.01).12

Then Eq. (11) can be written as

13 $$\left\{ \begin{aligned} & \Delta {\text{w}}_{{{\text{n}} + 1}} = \alpha \Delta {\text{w}}_{{\text{n}}} - \eta {\text{E}}_{{\text{w}}} \left( {{\text{w}}_{{\text{n}}} ,{\text{V}}_{{\text{n}}} } \right) \\ & \Delta {\text{v}}_{{{\text{n}} + 1}}^{{\text{i}}} = \alpha_{{{\text{n}},{\text{i}}}} \Delta {\text{v}}_{{\text{n}}}^{{\text{i}}} - \eta {\text{E}}_{{{\text{v}}_{{\text{i}}} }} \left( {{\text{w}}_{{\text{n}}} ,{\text{V}}_{{\text{n}}} } \right)\quad {\text{i}} = 1, \ldots , {\text{N}},\;\;{\text{n}} = 1,2, \ldots . \\ \end{aligned} \right.$$

The convergence of the adaptive momentum algorithm is said to be weakly convergent under the following assumptions.

- The denotation subset function ${\text{f}}\left( {\text{t}} \right)$, and their derivatives ${\text{f}}^{\prime } (t)$, and ${\text{f}}^{\prime \prime } {\text{(t)}}$ of Eq. (1) are uniformly bounded for all ${\text{t}} \in {\mathcal{R}}$
- ${\text{W}}_{{\text{n}}} { }({\text{n}} = 1,2, \ldots ..$ are uniformly bounded)
- The following set has a finite number of elements

14 $$\varphi = \left( {{\text{w}},{\text{V}}} \right){\text{|E}}_{{\text{w}}} \left( {{\text{w}},{\text{V}}} \right) = 0,\;{\text{E}}_{{\text{w}}} \left( {{\text{w}},{\text{V}}} \right) = 0,\;{\text{i}} = 1, \ldots .,{\text{ N}}\}$$

Assuming that the error function given by (12) and the weight sequence $\left\{ {{\text{W}}_{{\text{n}}} } \right\}$ generated by (13) with an initial weight value ${\text{W}}_{0}$ confirms that using assumption (a), (b), and (c) will hold for the final network output.

1. $${\text{E}}\left( {{\text{W}}_{{{\text{n}} + 1}} } \right) \le {\text{E}}\left( {{\text{W}}_{{\text{n}}} } \right),{\text{ n}} = 0,{ }1,{ } \ldots$$
2. There is ${\text{E}}^{*} \ge 0$ such that ${\text{lim}}_{{{\text{n}} \to \infty }} {\text{E}}\left( {{\text{w}}_{{\text{n}}} ,{\text{V}}_{{\text{n}}} } \right) = {\text{E}}^{*}$
3. $${\text{lim}}_{{{\text{n}} \to \infty }} {\text{E}}_{{\text{W}}} \left( {{\text{w}}_{{\text{n}}} ,{\text{ V}}_{{\text{n}}} } \right) = 0$$
4. $${\text{lim}}_{{{\text{n}} \to \infty }} {\text{E}}_{{{\text{v}}_{{\text{i}}} }} \left( {{\text{w}}_{{{\text{n}},}} ,{\text{ V}}_{{\text{n}}} } \right) = 0,\;{\text{i}} = 1,{ } \ldots .,{\text{ N }}$$

For any input  , the output of the hidden neurons is  , and the network output is15

Hence if assumption (c) is satisfied, then the (15) will converges to a local minimum $\left( {{\text{w}}^{*} ,{\text{V}}^{*} } \right)$, which means

16 $${\text{lim}}_{{{\text{n}} \to \infty }} {\text{w}}_{{\text{n}}} = {\text{w}}^{*} ,\;{\text{lim}}_{{{\text{n}} \to \infty }} {\text{V}}_{{\text{n}}} = {\text{V}}^{*}$$

17 $${\text{E}}_{{\text{w}}} \left( {{\text{w}}^{*} ,{\text{V}}^{*} } \right) = 0,\;\;{\text{E}}_{{{\text{v}}_{{\text{i}}} }} \left( {{\text{w}}^{*} ,{\text{V}}^{*} } \right) = 0,\;\;{\text{i}} = 1,{ }..,{\text{ N}}$$

The proposed variable momentum algorithm is different from previous state of the art methods as it uses the error to update the momentum term which means that the momentum term is directly related to the error value and behaves in such a way to reduce the error. In the next section, medial data preprocessing is going to be explained in which gets transformed, to bring it to such a state that the proposed variable adaptive momentum can easily parse it. In our experiments, 3 medical datasets have been selected to include all classification tasks: binary, multi label and multi class classification.

## Experimental study

### Brain tumor dataset

**Fig. 1:**
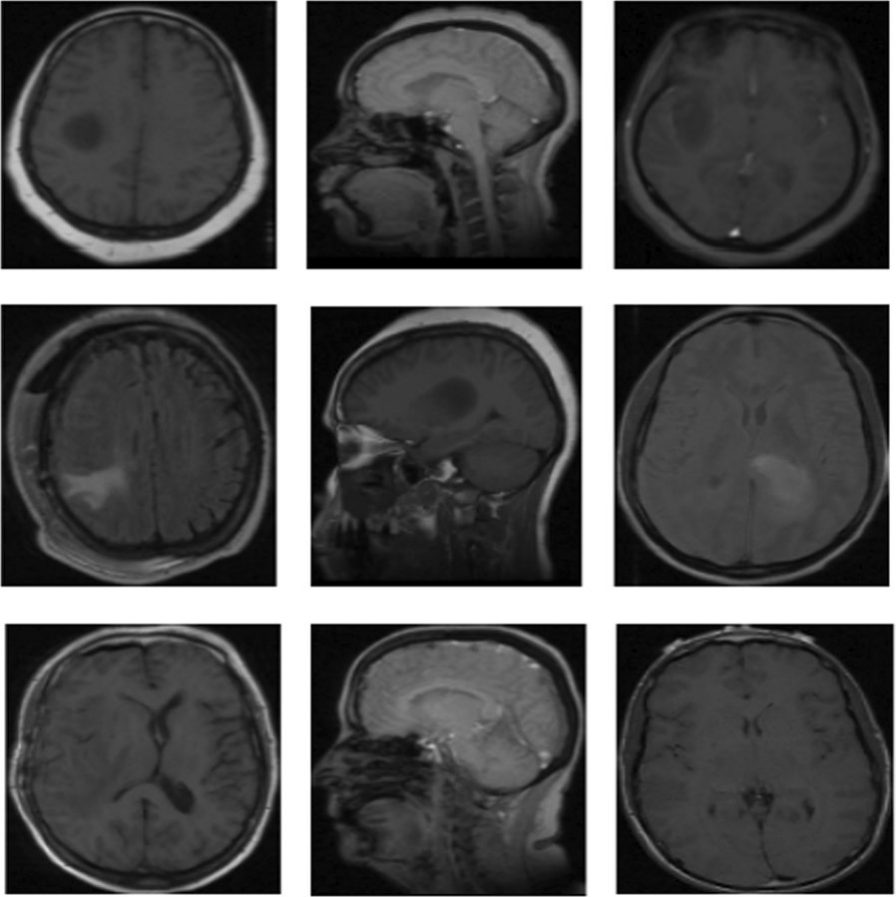
Samples from REMBRANDT brain tumor dataset

The largest cancer imaging archive from the REMBRANDT dataset for multi-class classification (Fig. 1) comprises 110.020 MRI images of tumors for 130 patients [27]. We only focused Astrocytoma, Glioblastoma, Oligodendroglioma and unidentified tumor image types. We have not considered the grade types of the tumor. In the experiments, we found out that, some patient's IDs were not found in metadata and some images were detected as outliers. We removed those images from the dataset. Finally, 106.541 images are classified for processing. The standard format for MRI images is DICOM image file format which was arranged according to the patient’s ID. In the next step, these images were converted into standard PNG format and were categorized based on types of tumors. This work was not done manually for all the images, but an automated approach is used with the help of metadata before conversion to PNG format. The study used encoded images which were represented by scalar string tensors.

### Chest X-ray dataset

**Fig. 2:**
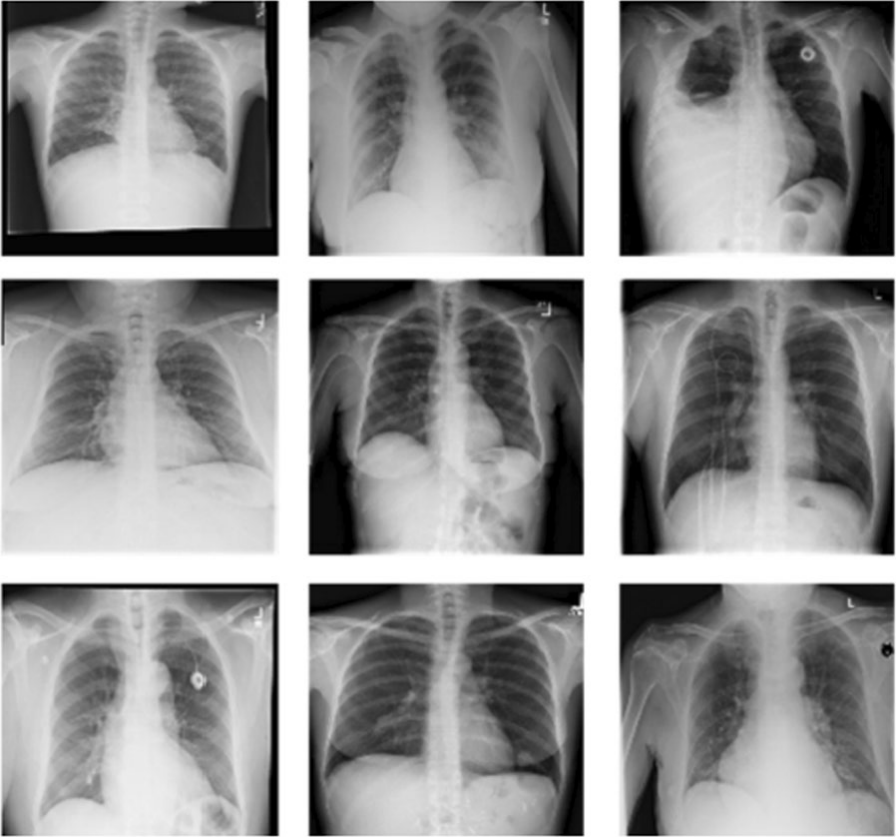
Samples from the NIH chest X-ray dataset

Chest X-ray examination is one of the most frequent and cost-effective medical imaging examinations. However, it may be difficult clinical diagnosis of chest X-rays and sometimes more difficult than diagnosing with a chest CT imaging. NIH Chest X-ray Dataset (Fig. 2) is comprised of 112,120 X-ray images with disease multi-labels from 30,805 unique patients [28]. The initial dataframe has been preprocessed to format (TFRecords) more suitable for CNN training purposes. Only images (downscaled to 600 × 600 and encoded as 1-channel jpegs) and corresponding diagnosis were left with all additional patient information excluded (e.g., age, sex, etc.). All 112,120 samples were kept (no filtration, grouping, or removing were performed). We focused all disease categories including Atelectasis, Consolidation, Infiltration, Pneumothorax, Edema, Emphysema, Fibrosis, Effusion, Pneumonia, Pleural Thickening, Cardiomegaly, Nodule, Mass and Hernia.

### Covid-19 CT scan dataset

**Fig. 3:**
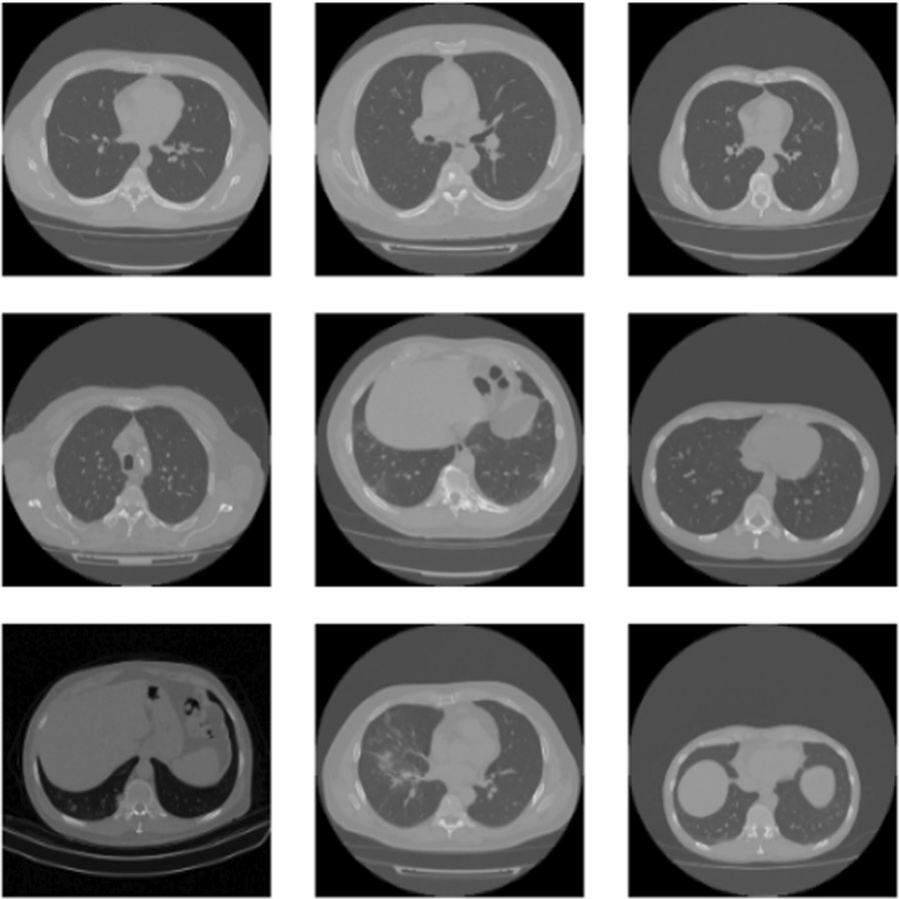
Samples from the Covid-19 CT scan dataset

During the pandemic, Maftouni [29] and his friends created a largest COVID-19 lung CT dataset (Fig. 3) so far, with 8.2 usability in Kaggle by curating data from 7 public datasets. These datasets have been publicly used in COVID-19 diagnosis literature and proven their efficiency in deep learning applications. Therefore, the merged dataset is expected to improve the generalization ability of deep learning methods by learning from all these resources together. The dataset has COVID-19, Normal, and CAP CT slices together with their corresponding metadata. Some of the datasets consist of categorized CT slices, and some include CT volumes with annotated lesion slices. Therefore, we used the slice-level annotations to extract axial slices from CT volumes. They converted all the images to 8-bit to have a consistent depth. They removed the closed lung normal slices that do not carry information about inside lung manifestations to ensure dataset quality. Additionally, they did not include images lacking clear class labels or patient information. In total, they have gathered 7,593 COVID-19 images from 466 patients, 6,893 normal images from 604 patients, and 2618 CAP images from 60 patients. To test all classification types in medical imaging, CAP labels are removed for this work to get the performance of binary classification. In our experiment, we only focused on patients who is diagnosed as Covid-19 and not Covid-19 for healthy ones.

### Data preprocessing and model simulations

During the preprocessing stage, CNN was subjected to image directly. The convolution kernel is then applied to pixel intensity in the image. The output of the convolution kernel is very dependent on the intensity values of the image. This intensity of pixels is not the same in all the images and it varied across images and subjects. The intensity of these images also depends on the image acquisition environment. These variations must be normalized for data mining approaches especially CNN. If the variations are not normalized it will result in a biased-conditioned network. The purpose of normalization to get the same range of values for different inputs into the CNN model. This helps in the stable convergence of the model. Therefore, in the preprocessing step, the intensity normalization is achieved using a minimum–maximum normalization approach. This scale the variable values to either [0,1]. Mathematically it is achieved using the following equation:

18 $${\text{y}}_{{\text{i}}} = {\text{ x}}_{{\text{i}}} { } - {\text{min}}\left( {\text{x}} \right)){ }/{\text{ max}}\left( {\text{x}} \right) - {\text{min}}\left( {\text{x}} \right)$$

where ${\rm{y}}_{\rm{i}}$ is the normalized intensity value against the position ${\rm{x}}_{\rm{i}}$ (i = 1.. n). The min(x) and max(x) represent the minimum and maximum intensity values for intensity in the entire image. Images were normalized and then resized. It was triplicated for creating three channels as per the prerequisite for the sized input model. The result of intensity normalization is that it generates the intensity which is in a coherent range across all images. This aids in the learning process of CNN. Reducing the image size reduces the memory requirements while increases the speed of the training process.

We used the cropping technique for brain tumor images due to feeding the model only with the brain image. In order to crop the part that contains only the brain of the image, we used a contour detection algorithm [36] to find the extreme points of the brain image.

In brain tumor and chest diagnosis data, we converted data structure hierarchy into a byte stream for binary serialization format such as Pickle and TFRecords, whereby a binary file is easily be converted back into an object hierarchy.

Neural networks need to incorporate non-linearity in their layers as it performs complex tasks. A ReLu activation function computes the output of each layers because other functions like Sigmoid requires much more calculation to find its gradient. ReLU on the other hand directly gives the gradient with less computation. This helps during backpropagation saving a lot of time and gradient computation power. One other reason is the gradient with sigmoid function saturates on having high or low numbers making it difficult to change the new weights. ReLu has a linear function for x > 0 which achieves no saturation.

19 $${\text{f}}\left( {\text{x}} \right) = {\text{max}}\left( {0,{\text{x}}} \right)$$

All neurons in the convolutional and fully connected layers use (3) and (4) to calculate the input and produce output. It is well achieved that, adding strides instead of pooling layer increases accuracy [37]. However, we used max pooling layer too between last 2 convolution layer to reduce network parameters low. The final layer computes the classification probability of each classification type using the Softmax function:

20 $$\sigma \left( {{\text{z}}_{{\text{i}}} } \right) = { }\frac{{{\text{e}}^{{{\text{z}}_{{\text{i}}} }} }}{{\mathop \sum \nolimits_{{{\text{j}} = 1}}^{{\text{K}}} {\text{e}}^{{{\text{z}}_{{\text{j}}} }} }}\;{\text{for}}\;{\text{i}} = 1, \ldots ,{\text{K}}\;{\text{and}}\;{\text{z}} = \left( {{\text{z}}_{1} , \ldots ,{\text{z}}_{{\text{K}}} } \right) \in {\text{R}}^{{\text{K}}}$$

As these processes includes all classification task, in the output node, we used cross-entropy to calculate loss,

21 $${\text{C}}\left( {{\text{x}},{\text{y}}} \right) = { } - \mathop \sum \limits_{{\text{i}}}^{{\text{c}}} {\text{y}}_{{\text{i}}} \times { }\log \left( {{\text{x}}_{{\text{i}}} } \right)$$

The model was simulated using Google Colab TPU and NIVIDA GPU Tesla K80 with 13 GB of memory. The model layers were tuned through an extensive set of experiments. We created sequential object and started off with a CNN layer. We set filters to 32, 64 and 128 for each 3 CNN layer respectively. We set the kernel size to 3 by 3 with same padding. We used 2 by 2 stride only in the second CNN layer. In other layer, stride parameters set by default 1 and a very common form of max pooling layer with filter size 2 × 2 applied. Passing a dense layer, we added 512 neurons.

The proposed method custom model is illustrated in Table 1, to reduce overfitting and stabilize learning process, we used dropout and batch normalization (BN). For the dropout (DO), we started with 0.3 and, we used 0.5 before dense layer. We used L2 regularization (weight decay) which adds squared values of weights in the cost function. We expected to see even the training accuracy gets worse, test performance will get better by diffusing weights. The custom model regularization hyperparameter is 0.0005. The visualization of the structure of the proposed model is shown below. Note that, model output shapes differ in each dataset due to input shapes of images.

**Table 1:** Example of model architecture and parameters

Layer	Output shape	Total parameter
Convolution	64 $\times$ 64 $\times$ 32	896
Batch Norm	64 $\times$ 64 $\times$ 32	128
Dropout	64 $\times$ 64 $\times 32$	0
Convolution	62 $\times$ 62 $\times$ 64	18.496
Batch Norm	62 $\times$ 62 $\times$ 64	256
Max Pool	15 $\times 15 \times 64$	0
Convolution	15 $\times 15 \times 128$	73,856
Batch Norm	15 $\times 15 \times 128$	512
Flatten	28,800	0
Dense	512	14.746.112
Batch	512	2048
Dropout	512	0
Dense	4	516

**Fig. 4:**
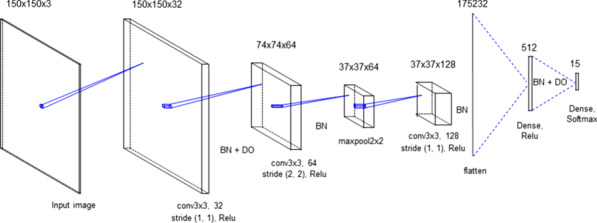
Proposed CNN Architecture

The purpose of the create and use a custom model (Fig. 4) is to reduce training time but during experiments, we tested our method with pre-trained models too which will be discussed in later sections.

## Evaluation results

### Performance metrics

The performance of the CNN image-based classification is evaluated for image-based classification for the parameters used in the confusion matrix, such as specificity, recall/sensitivity, accuracy, F1 score, and precision. These metrics are evaluated using the terms provided in Table 2.

$${\text{Accuracy}} = \frac{{{\text{TP}} + {\text{TN}}}}{{{\text{TP}} + {\text{FP}} + {\text{FN}} + {\text{TN}}}}$$

$${\text{Precision}}\;\left( {{\text{PPV}}} \right) = \frac{{{\text{TP}}}}{{{\text{TP}} + {\text{FP}}}}$$

$${\text{Recall}}\;\left( {{\text{Sensitivity}}} \right) = \frac{{{\text{TP}}}}{{{\text{TP}} + {\text{FN}}}}$$

$${\text{F}}1\;{\text{Score}} = 2*\frac{{{\text{Precioson}}*{\text{Recall}}}}{{{\text{Precision}} + {\text{Recall}}}}$$

The confusion matrix parameters of Rembrandt dataset’s TP result is 56128, TN is 45285, FN is 1064 and FP is 4064. Chest X-Ray dataset’s TP result is 83652, TN is 12368, FN is 5095 and FP is 11005. Covid 19 dataset’s TP result is 6060, TN is 1020, FN is 420 and FP is 93.

**Table 2:** Defining the terms TP, FP, FN, TN

Predicted label	Actual Label	Definition
Positive	Positive	True positive (TP)
Positive	Negative	False positive (FP)
Negative	Positive	False negative (FN)
Negative	Negative	True negative (TN)

### Experiment results

We split the Chest X-Ray and Covid-19 images into train and test with a ratio of 20%-80% and 25%-75% for brain tumor image by random sampling to assess the classification performance of the proposed model. The training accomplished using several Python libraries such as Scikit-learn, and TensorFlow. We reduced the images size to limit memory consumption per iteration. We tested our method with well-known optimizers such as Adam, RMSprop and SGD. We used standard parameters (${\beta }_{1}$ = 0.9, ${\beta }_{2}$ = 0.999) a learning rate of $\alpha$ = 0.001 for Adam and RMSProp with $\varepsilon =1e-07$, and $\alpha$= 0.01 for SGD and proposed method. To test fast convergence of the proposed model, the data trained 5 epochs to experiment validation accuracy with custom model. The accuracy comparison with different optimizers in different datasets are shown in Tables 3, 4 and 5.

**Table 3:** Accuracy comparison among the proposed method on REMBRANDT brain tumor dataset

Epoch	Adam	RMSprop	SGD	Adaptive momentum
1	0.72	0.70	0.70	0.73
2	0.79	0.76	0.77	0.83
3	0.81	0.79	0.82	0.88
4	0.81	0.80	0.85	0.90
5	0.83	0.80	0.88	0.91

**Table 4:** Accuracy comparison among the proposed method on NIH chest X-ray dataset

Epoch	Adam	RMSprop	SGD	Adaptive momentum
1	0.83	0.80	0.84	0.82
2	0.84	0.76	0.83	0.83
3	0.84	0.84	0.84	0.84
4	0.83	0.79	0.83	0.85
5	0.79	0.83	0.84	0.85

**Table 5:** Accuracy comparison among the proposed method on Covid-19 dataset

Epoch	Adam	RMSprop	SGD	Adaptive momentum
1	0.85	0.58	0.90	0.87
2	0.89	0.93	0.91	0.90
3	0.84	0.93	0.93	0.92
4	0.92	0.82	0.94	0.82
5	0.93	0.48	0.93	0.92

Our experimental results demonstrated that proposed adaptive momentums converges better than Adam, RMSprop and SGD in brain tumor and chest x-ray dataset. As shown in Table 5. RMSprop is more locally unstable. We suspect that this is the case because we used small batch size (32) and trained large network with working small size dataset as it can cause fluctuations. As shown in Table 6 the proposed model achieved 95% F1 score for multi class classification, 85% for multi label classification and 93% for binary classification. Despite an unequal distribution of classes for brain image and chest x-ray datasets, the weighted and macro averages of the precision and recall scores are promising.

**Table 6:** Classification results of proposed model

Tumor type	Precision	Recall	F1 score
REMBRANDT	0.94	0.97	0.95
NIH chest X-ray	0.83	0.84	0.85
Covid-19	0.94	0.92	0.93

Table 7 describes the comparison of proposed approach with state of the art methods on different medical datasets. The author [38] used hybrid genetic algorithm and particle swarm optimization (PSO) with 62% accuracy achievement whereas other author [39] used firefly algorithm and adopted tolerance rough ret (TRS) and achieved accuracy of 90%. All the results are taken from the original paper [40]. The proposed method achieved the highest classification accuracy of 95%. When comparing with Covid-19 studies [29] we want to indicate, authors trained the networks for 50 epochs whereas we only trained 5 epochs as it converges 10 times faster.

**Table 7:** Accuracy comparison among the proposed and state of the art methods

REMBRANDT	HGAPSO [38]	FATRS [39]	Proposed method
Test accuracy	0.62	0.90	0.95
NIH chest X-ray	GAC [41]	DNT [42]	Proposed method
Test accuracy	0.84	0.60	0.85
Covid-19	FC [29]	DN [29]	Proposed method
Test accuracy	0.95	0.92	0.93

To strengthen the outcomes of this study we investigated the performance comparison for the state of the art CNN architectures using the proposed method. The usefulness of transfer learning and fine-tuning for smaller datasets have been proposed on [21] but for experimenting with big datasets are still time consuming.

We compared the classification performance of the most well-known 3 pre-trained CNN models (i.e., Xception, Resnet50 and VGG16) trained on Imagenet [43]. The Xception model has 71 deep layers and proposed by Francois Chollet [44]. Resnet50 has 50 deep layers [45] while VGG16 (also called Oxfordnet) is proposed by [46] and is a convolutional neural network that has 16 layers deep.

## Discussion

**Fig. 5:**
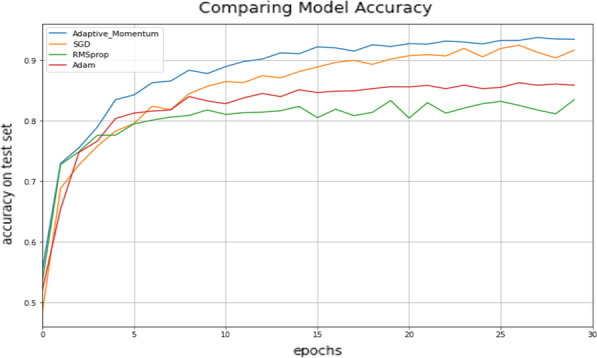
Convergence curve of fine-tune process

Adaptive momentum algorithm, which is directly related to error variation, converges much faster to a minimum compared to the conventional optimizers. Table 8, shows the comparison results between the proposed method and pre-trained models. The results were achieved after five epochs; the model execution time reduced 60% when compared with pre-trained models. Pre-trained models have approximately 20 times much more parameters to train. We confirm that parameter tuning is time-consuming, even for GPUs and TPUs. Our experiments created a simple CNN model, altered the conventional training method, and included the modified momentum term to get sub-optimal parameters for neurons. The proposed method convergence speed is 20% higher than conventional SGD. During the fine-tuning experiments, the convergence capability of the proposed method and the best test accuracy of the fine-tuning process are shown in (Fig. 5).

**Table 8:** Accuracy comparison of pre-trained CNN models with proposed method on different medical image datasets

Dataset	Xception	ResNet50	VGG16	Proposed model
REMBRANDT	0.87	0.91	0.94	0.95
Chest X-ray	0.83	0.84	0.88	0.85
Covid-19	0.96	0.98	0.52	0.93

The pre-trained models with custom adaptive momentum were tested during the experimental process. Even though the proposed method achieved 0.97% accuracy compared to Xception which, achieved 87% accuracy [29] for the Covid-19 dataset, the accuracy of VGG16 and Resnet50 did not change. The test results show that both the VGG16 and Resnet50 have performed slightly better than the proposed method. Considering that the proposed method training dataset size and number of training epochs are small compared to the pre-trained models, it can be said that the proposed method has performed very well against both of these pre-trained models. This type of comparison will not be considered as a fair comparison since the pre-trained models were trained on millions of images for a much higher number of epochs to achieve this type of result.

There are three major obstacles in using medical images to train algorithms: the class imbalance challenge, the multitask challenge, and the dataset size challenge. In this work, several techniques are presented to tackle them. We used different class weights for each class and passed it to the CNN for the class imbalance challenge. Another challenge that we encounter in the medical image classification setting is the multitask challenge. It is complex and challenging without having underfitting. To overcome this, we used dropout and batch normalization techniques. To train such an algorithm, it was also needed to modify the loss function from the binary tasks to the multitask setting. TPUs and GPUs were used with high RAM in Google Colab; this helped to ease the dataset size challenge. The convolutional neural network is used as the default architecture for many medical imaging problems. These are designed to process 2D images like x-rays. But variants of these are also well suited to medical signal processing or 3D medical images like CT scans, which we will look at in our future works. The standard is to try out multiple models on the desired tasks in medical problems and see which ones work best.

Our future aim is to automate the learning rate depending on the loss value. We will define a function that returns the new learning rate as output in each epoch. This function will be controlled by loss values and either increase or decrease within the specified range. We will create a learning rate scheduler instance and pass the previously defined function as a parameter.

## Conclusion

This paper presents an adaptive algorithm in which the adaptive term is directly related to error variation; the algorithm updates the weight vector according to the input vector. Therefore, the algorithm is controlled by the learning rate parameter, which depends on the eigenvalues of the input's autocorrelation matrix, resulting in an improvement in the backpropagation algorithm compared to the conventional algorithm. The results show that the proposed algorithm achieved faster convergence behavior and minimized the miss adjustment error in the steady-state optimum solution.

The proposed method improves SGD performance by reducing classification error from 6.12 to 5.44%, and it achieved the lowest error and highest accuracy compared with other optimizers. Testing results of Table 7 show that the proposed algorithm achieved 95%, 85%, and 93% testing accuracy against the brain tumor, chest x-ray, and covid-19 datasets, respectively. Compared to the best benchmark pre-trained models (Xception, ResNet50 and VGG16), we achieved best results in REMBRANDT an Chest-X-Ray dataset with 60% fast execution time. The performance of the proposed method against the other two pre-trained methods was acceptable, meaning that the proposed method can achieve even better results if the training conditions are changed to be similar to that of the pre-trained methods.

The proposed algorithm performance was further compared against a conventional SGD based algorithm; the proposed algorithm achieved accuracy results of 95% while the result of the conventional SGD is 92%, resulting in convergence speed is 20% higher than the conventional SGD. The result shows that the tuning time required to tune the adaptive term was negligible. The results also confirm that the proposed algorithm can perform well against unbalanced datasets since the dataset chosen for the testing are all highly unbalanced datasets.

## Data Availability

The datasets analyzed in this paper are publicly available. All the datasets used is this paper are referenced directly via listing in the references. Also, the repository of the research can be found in https://github.com/utkucanaytac/Adaptive_Momentum_
